# Early teacher identity, social empathy, and inclusive efficacy: a PLS-SEM investigation among pre-service teachers

**DOI:** 10.3389/fpsyg.2026.1854575

**Published:** 2026-07-02

**Authors:** Zehra Yaşar Sağlık, Esra Akgül

**Affiliations:** 1 Department of Primary Education, Faculty of Education, Hasan Kalyoncu University, Gaziantep, Türkiye; 2 Department of Early Childhood Education, Faculty of Education, Hasan Kalyoncu University, Gaziantep, Türkiye

**Keywords:** early teacher identity, inclusive efficacy, PLS-SEM, pre-service teachers, social empathy

## Abstract

**Introduction:**

This study examined the relationships among early teacher identity, social empathy, and teacher efficacy for inclusive practices among pre-service teachers.

**Methods:**

A quantitative, cross-sectional survey design was employed. The study sample consisted of 510 undergraduate pre-service teachers (267 early childhood education and 243 primary education students) from seven Turkish universities during the 2025–2026 academic year. Data were collected using the Attitude toward Teaching Profession Scale, the Social Empathy Scale, and the Teacher Efficacy for Inclusive Practices Scale (TEIP). Data analysis was conducted using Partial Least Squares Structural Equation Modeling (PLS-SEM) with SmartPLS 4.0. The analysis followed a two-stage assessment of measurement and structural models and included a 5,000-resample bootstrapping procedure to test the hypothesized relationships.

**Results:**

Findings revealed that early teacher identity was significantly and positively associated with both social empathy and teacher efficacy for inclusive practices. In addition, social empathy demonstrated a statistically significant indirect effect in the relationship between early teacher identity and teacher efficacy for inclusive practices. The model explained 43.3% of the variance in TEIP.

**Discussion:**

The results suggest that teacher education programs may benefit from greater emphasis on identity development and socio-emotional competencies, particularly social empathy, in preparing pre-service teachers for inclusive education.

## 1 Introduction

The examination of the relationships among Early Teacher Identity (ETI), Social Empathy (SE), and Teacher Efficacy for Inclusive Practices (TEIP) in pre-service teachers holds multidimensional significance in explaining and improving the quality of inclusive education. In contemporary educational policies, inclusive education is addressed not merely as a pedagogical approach, but as a rights-based paradigm grounded in equality, participation, and respect for diversity. In this context, teacher education is considered one of the most critical leverage points for the systemic transformations required to expand inclusive education (European Agency for Development in Special Needs Education, 2012). Within this framework, the success of inclusive education is largely associated with teachers’ professional identities and their perceptions of competence. Beliefs about teaching efficacy, which constitute a significant component of teachers’ professional identities, are strong predictors of the extent to which they implement inclusive classroom practices. It is noted that teachers with high efficacy beliefs tend to support student achievement, foster self-confidence, and employ flexible instructional strategies, whereas those with low efficacy beliefs tend to perceive differences as deficiencies and focus more on classroom management. Therefore, understanding how pre-service teachers’ philosophical orientations toward inclusive education are related to their perceived readiness for inclusive classroom practices necessitates a holistic examination of their professional identity and efficacy beliefs (Woodcock et al., 2022).

However, inclusive education is not limited to cognitive or pedagogical competencies; it also encompasses strong affective and relational dimensions. At this point, empathy emerges as a fundamental prerequisite for establishing inclusive classrooms and reducing prejudice. Inclusive education requires not only physical placement but also an understanding that views diversity as a valuable resource, which is directly related to individuals’ levels of empathy (Kudrnáč et al., 2024). Empathy is generally understood as a multidimensional capacity that enables individuals to understand and respond to others’ emotions, needs, and experiences. In educational contexts, empathy may foster social relationships and may contribute to more positive attitudes toward disadvantaged groups; however, its effects may vary depending on the specific dimension of empathy considered (Kudrnáč et al., 2024). Cognitive empathy and affective empathy are well-distinguished constructs in educational research. It is consistently defined as the capacity for perspective-taking and understanding others’ mental states (Bezzina, 2022; Tikkanen et al., 2022), while affective empathy refers to emotional sharing, mirroring, or resonance with another’s feelings (Domínguez-Arriola, 2025; Feshbach and Kuchenbecker, 1974). These two constructs are supported by doubly dissociable brain systems (Buck and Xu, 2020), show developmental and psychological independence (Maxwell and DesRoches, 2010), and are measured by distinct subscales on established instruments such as the TECA and IRI (Domínguez-Arriola, 2025). Social empathy, by contrast, does not emerge as a comparably formalized construct in the educational literature reviewed. The closest analogue is Feshbach and Kuchenbecker’s concept of “social comprehension,” described as necessary but not sufficient for empathy and associated with academic progress (Feshbach and Kuchenbecker, 1974), but this has not been consolidated into a widely adopted category in subsequent educational research. Although affective empathy is highly relevant in educational settings, the present study focuses specifically on social empathy because the selected measure captures the cognitive-contextual understanding of others’ lived experiences rather than emotional sharing or affective resonance. Therefore, the findings should be interpreted in relation to the social-cognitive and contextual dimensions of empathy rather than affective empathy. Accordingly, it is important to examine the association between pre-service teachers’ levels of empathy and their perceived competencies for inclusive education. In particular, the fact that SE extends beyond individual empathy to encompass intergroup sensitivity necessitates a more comprehensive consideration of this relationship.

The culturally sensitive pedagogical competence of teachers has crucial potential for eliminating educational inequalities, prejudices, and biases (Celik et al., 2025). It is increasingly emphasized that teacher competencies are not limited to knowledge and skills but also encompass attitudes, beliefs, and values. Inclusive education is shaped not only by technical pedagogical skills but also by core values such as equality, participation, and respect for diversity. The “Profile of Inclusive Teachers” developed at the European level also demonstrates that teacher competencies include affective and value-based dimensions in addition to cognitive and practical aspects (European Agency for Development in Special Needs Education, 2012). Similarly, UNESCO (2020) states that one of the most significant barriers to inclusive education is the lack of belief that it is both feasible and desirable. Therefore, examining how pre-service teachers’ ETI, SE and TEIP are reflected in self-reported beliefs and perceived competencies related to inclusive practices is important for understanding the structural barriers in inclusive education.

On the other hand, the nature of these relationships and their underlying mechanisms have not yet been sufficiently clarified in the literature. Although the strong relationship between teachers’ self-efficacy beliefs and inclusive practices is widely acknowledged, the question of which psychosocial variables account for these differences remains unanswered (Woodcock et al., 2022). In particular, there is a need for more in-depth research to determine whether these differences stem from pre-service teacher education processes or from individual characteristics, such as levels of empathy (SE). Moreover, studies on empathy have largely focused on interpersonal relationships and prejudice, while its role within the context of inclusive education has been addressed only in a limited manner (Kudrnáč et al., 2024). This theoretical gap becomes even more critical in the context of early childhood and the first years of primary education. These periods represent stages in which learning occurs most rapidly and development is most sensitive. Indeed, a child’s brain reaches approximately 80% of its adult size by the age of three, and synaptic development continues intensively until around the age of eight (Campana et al., 2024). This situation highlights sensitive periods, defined as “windows of opportunity,” during which language, social–emotional development, and executive functions are acquired most effectively (Varga, 2017). Teachers who interact with children during these sensitive periods influence not only learning processes but also developmental structures in a lasting way. The strong relationship between teachers’ educational levels and professional competencies and children’s developmental outcomes (Mayfield et al., 2006), as well as the role of teacher–child interactions, clearly demonstrate the critical position of teachers working in this period. In this context, it is necessary to examine more deeply the individual and professional characteristics (e.g., SE and TEIP) of pre-service teachers who will work in early educational settings. Therefore, the present study focuses on pre-service preschool and primary school teachers, who play a critical role in early childhood and early primary education contexts.

**Figure 1:**
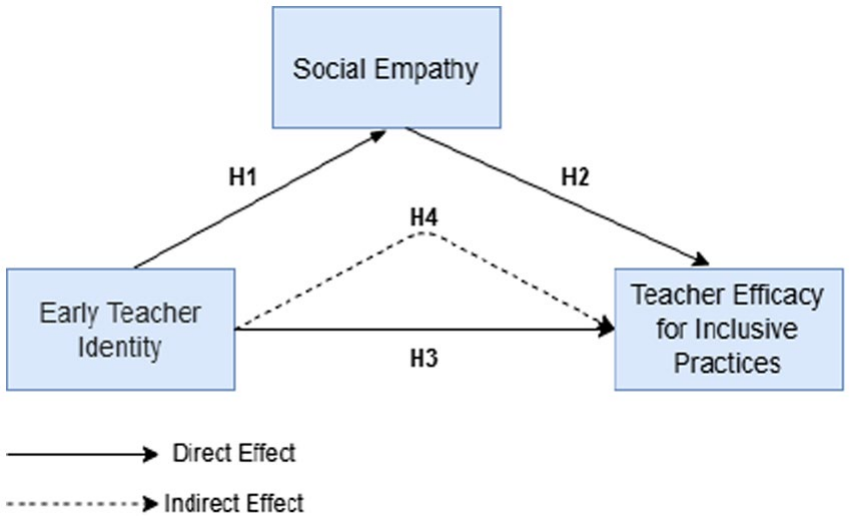
Hypothetical model.

In conclusion, the most critical responsibility for the effective implementation of inclusive education for all students rests with teachers and teacher education systems (UNESCO, 2020). In this regard, examining the relationships among pre-service teachers’ levels of SE, ETI, and TEIP within a holistic model is of great importance for restructuring teacher education programs in line with the ideal of “education for all” (European Agency for Development in Special Needs Education, 2012; Forlin et al., 2011). In this context, the model proposed in the study is presented in Figure 1, and the research hypotheses developed based on this model are provided below.

Hypotheses:

*H1*. ETI is positively and significantly associated with SE.

*H2*. SE is positively and significantly associated withTEIP.

*H3*. ETI is positively and significantly associated with TEIP.

*H4*. SE statistically accounts for an indirect association between ETI and TEIP.

### 1.1 Theoretical and conceptual framework

Early Teacher Identity (ETI) is a dynamic, multifaceted and contextually situated process of professional self-construction, with relational, personal and professional aspects (Morrison, 2013; Pearce and Morrison, 2011; Seward and Renwick, 2013). It is broadly defined as how people perceive and understand themselves as educators (Horn et al., 2008) and is influenced by social interactions and institutional discourses (Trent, 2016), mediated by reflection and metacognitive awareness (Erdem, 2020), narrative construction (Pearce and Morrison, 2011) and the development of expected and feared possible selves (Erdem, 2020). It is a construct that from early self-categorization in pre-service education (Erdem, 2020) to the first years of professional practice (Trent, 2016) is characterized by intense self-evaluation (Morrison, 2013), idealism-reality gap (Pearce and Morrison, 2011) and the reconciliation of conflicting identities (Babanoglu and Agcam, 2019). Morrison (2013) articulates three identity paths, emergent, tenuous and distressed, that connect social and institutional support to teachers’ professional stability or early departure. Pearce and Morrison (2011) note that “intentional, relational engagement in identity development enhances resilience” (p. 5). Early teacher identity is a tangible concept that affects teacher attrition.

Second, according to Segal (2011) and Segal et al. (2012), Social Empathy (SE) is most often defined as the ability to understand others by appreciating their life circumstances and gaining insight into institutional disparities and injustices. The dominant conceptual model, proposed by Segal and colleagues, consists of three central components: contextual understanding of systemic barriers, macro-level perspective-taking or social responsibility, and interpersonal empathy (which encompasses affective response, cognitive processing, self-other awareness, perspective-taking, and emotion regulation) (Gerdes and Segal, 2009; Segal, 2011; Segal et al., 2012). These elements have been empirically validated by the Social Empathy Index (Segal et al., 2012). It is hypothesized that they are hierarchical, with interpersonal empathy being the base for contextual and macro-level aspects.

Finally; Teacher Efficacy for Inclusive Practices (TEIP) is a construct that captures teachers’ confidence in their ability to successfully implement inclusive education in their classrooms, based on Bandura’s self-efficacy theory (Dias, 2017; Dignath et al., 2022; Martins and Chacon, 2021). It is operationalized through three main aspects: effectiveness in controlling student behavior, effectiveness in working with families and professionals, and effectiveness in inclusive instruction (Dias, 2017; Ozokcu, 2018; Selin Tumkaya and Miller, 2020). TEIP is conceptually different from general teaching efficacy (Dignath et al., 2022; Shaukat, 2012) as it underpins the specific challenges of inclusive environments such as adapting curricula for diverse learners and engaging with experts. The TEIP was found to be positively correlated with attitudes toward inclusive education (Ozokcu, 2018) and importantly differentiated teachers based on their enacted practices rather than their conceptual understanding of inclusion (Woodcock et al., 2022). Demographic factors such as gender are weak and inconsistent predictors of TEIP (Selin Tumkaya and Miller, 2020) whereas special education training (Dignath et al., 2022), direct contact with students with disabilities (Dias, 2017; Ozokcu, 2018) and practicum length (Charles et al., 2025) are the strongest predictors.

### 1.2 The formation of ETI and SE in the context of inclusive education

In inclusive education, teacher roles have been reshaped over the past three decades through structural and pedagogical transformations at the international level, shifting from individually oriented practices toward collaborative and participation-based professional practices (Keeffe and De George-Walker, 2010). This transformation represents a multilayered process that directly influences not only instructional practices but also the construction of teacher identity. Indeed, the transition of special education teachers from independent resource rooms to collaborative consultation models within general education classrooms (Klingner and Vaughn, 2002), along with the shift of general education teachers from teacher-centered approaches to pedagogies prioritizing student participation and inclusive learning environments (Schwab et al., 2015), constitute concrete reflections of this professional repositioning. Within this process, teachers have moved away from evaluating students’ difficulties solely within a deficit-based framework and have instead adopted a more holistic and participation-oriented professional perspective centered on the question, “How can I increase student participation?” (Van De Putte and De Schauwer, 2013). This transformation has been shaped by the interaction of policy-driven regulations, increasing student diversity, and shifts in pedagogical paradigms; consequently, teachers are expected to demonstrate advanced professional competencies in areas such as co-teaching, curriculum adaptation, and collaborative planning (Antia, 1999). Therefore, teaching in inclusive education settings requires not only the application of general pedagogical skills but also the development of flexible, adaptive, and context-sensitive strategies for students with diverse learning needs (Pickl et al., 2016). At this point, teacher competencies extend beyond technical skills and become one of the fundamental determinants structuring the early formation process of ETI.

ETI is largely shaped through pre-service teachers’ attitudes, beliefs, and self-efficacy perceptions developed during their education process. However, research indicates that initially positive attitudes toward inclusivity among pre-service teachers may weaken during the later stages of their education if sufficient supportive structures are not provided. In particular, when a consciously structured inclusive education experience is not offered, encountering the realities of teaching practice may lead to a decline in motivation (Costello and Boyle, 2013). This situation demonstrates that the formation of ETI is determined not only by theoretical knowledge transfer but also by structured experiential learning processes. In this framework, teachers’ self-efficacy perceptions regarding inclusive education practices are directly dependent on the quality of their preparation programs, and significant international variations exist in this regard. Comparative studies conducted with samples from Canada, Australia, Hong Kong, and Indonesia have identified the structure of teacher education programs, knowledge of inclusion policies, prior interactions with individuals with disabilities, and the extent to which programs include inclusive practices as key determinants (Loreman et al., 2013). These findings show that ETI is shaped not only by individual dispositions but also by systemic educational policies.

In parallel, the higher self-efficacy perceptions reported by pre-service teachers enrolled in dual certification programs highlight the importance of program type in early identity development. However, the limited opportunities to practice inclusive teaching strategies may partially weaken this positive effect. On the other hand, it has been found that extended practicum experiences in inclusive settings strengthen self-efficacy over time, and particularly that prior beliefs supporting inclusion further reinforce this development (Charles et al., 2025). This indicates that ETI is constructed not only as a cognitive process but also as an experiential one.

The insufficient development of ETI can directly lead to negative outcomes in inclusive education. Indeed, inadequate teacher preparation is associated with a wide range of problems in students with disabilities, including lower academic achievement, increased social isolation, reduced self-confidence, and experiences of bullying (Nomtshongwana et al., 2025). In this context, deficiencies in teacher education, ineffective instructional practices, and negative teacher attitudes emerge as major barriers to inclusive education (Grant and Jones-Goods, 2016). Moreover, the persistence of deficit-based approaches and the insufficient development of differentiated instruction skills are among the key structural problems that limit the formation of early professional identity (Arnaiz-Sánchez et al., 2023).

As a result, within the context of inclusive education, ETI is constructed through an interactive process between teacher competencies and pre-service teachers’ educational experiences. This process is not limited to individual development; it is also shaped by teacher education policies, program structures, and practical training opportunities. Therefore, strengthening ETI is considered a critical prerequisite for the sustainability of inclusive education.

In this regard, another key variable that shapes pre-service teachers’ inclusive education competencies is SE. This is because ETI is not only related to professional role perceptions and pedagogical competencies but may provide a conceptual foundation for understanding how pre-service teachers position themselves in relation to students’ experiences, needs, and classroom diversity. SE is a multidimensional construct that involves not only understanding others’ emotions and thoughts but also comprehending the social, economic, and historical contexts in which these experiences are embedded. This concept, developed by Elizabeth Segal, focuses on understanding the living conditions of individuals from different socioeconomic and ethnic/racial groups and developing awareness of the structural dynamics that shape these conditions (Segal, 2011; Segal et al., 2012). In this respect, SE extends traditional notions of empathy beyond the level of individual interaction and offers a more comprehensive framework that includes the analysis of social structures (Hylton, 2018).

This theoretical expansion demonstrates that SE is not merely an affective response but also a cognitive and critical awareness process. Accordingly, the concept is positioned as a holistic competence that requires individuals to simultaneously evaluate both micro-level interpersonal relations and macro-level social inequalities. Within this framework, the SE model is structured around three core components.

First, interpersonal empathy constitutes the affective and cognitive foundation of the model. This component includes affective responsiveness to others’ emotions, maintaining the distinction between self and other, perspective taking, and emotional self-regulation skills. At this level, empathy represents the most fundamental layer of the empathic process operating in interpersonal interaction.

However, interpersonal empathy alone is insufficient to explain social reality. Therefore, the second component, contextual understanding of systemic barriers, extends empathy to a structural level. This component requires the analysis of historical processes, economic inequalities, and institutionalized mechanisms of discrimination that shape the living conditions of different social groups (Segal et al., 2012). In this way, individuals are able to interpret differences not only at the individual level but also within the context of structural production processes.

The third component, macro-level self–other awareness and perspective taking, represents the most comprehensive cognitive dimension of SE. This component refers to the ability of individuals to transcend their own social position and understand the experiences of different social groups, even without directly experiencing them, and to establish an empathic connection with these experiences (Hylton, 2018; Segal et al., 2012).

This theoretical structure is also supported by findings in the context of inclusive education. Research shows that empathy levels are associated with more positive attitudes toward peers with special needs and higher levels of prosocial behavior (Ling et al., 2025; Lombardi et al., 2015; Löper et al., 2026). These findings indicate that SE is not only an individual trait but also an important psychosocial variable that determines social acceptance and the quality of interaction in inclusive educational environments.

### 1.3 ETI, SE and TEIP: a relational perspective

ETI, SE, and TEIP are conceptualized as interrelated core structures that mutually reinforce one another in the development of the teaching profession. In this context, empathy and professional identity play a role in both the cognitive and affective dimensions of the teaching process.

The relationship between early professional identity formation and empathy in teaching has received increasing attention in the literature. Studies conducted with preschool teachers demonstrate that teacher identity and levels of empathy have significant effects on professional processes (Chen et al., 2025). Similarly, it has been reported that teachers’ professional identities significantly and positively predict empathy (Sun et al., 2025). Ince and Coskuner (2025) revealed that life skills such as empathy, self-awareness, and communication are strong determinants of teacher identity. In addition, Goroshit and Hen (2016) found that teacher self-efficacy predicts empathy, suggesting that efficacy-based self-beliefs may play a mediating role in this relationship. Mchenry and Kelly (2023), through a qualitative study, showed that pre-service teachers participating in cultural responsiveness programs developed empathy through social engagement and shifted from a deficit-oriented perspective to an asset-oriented perspective. Given the close relationship between empathy and SE, it is suggested that ETI may positively influence SE.

SE may be considered an important socio-emotional correlate of TEIP. The literature emphasizes that empathic teachers are able to create supportive learning environments for diverse learning needs, use differentiated instruction strategies more effectively, and enhance student motivation and engagement (De Klerk and De Klerk, 2018; Gómez-Arizaga et al., 2016). In addition, empathy has been associated with the use of active teaching methods, inclusive planning, and professional development processes (Orozco and Moriña, 2023). Empathy facilitates the understanding of students’ individual experiences and contributes to the development of trust-based relationships between teachers and students (Villena-Martínez et al., 2024). Moreover, a linear relationship has been reported between students’ perceived empathic classroom climate and their self-esteem; students with low self-esteem who lack an empathic environment may resort to bullying behaviors in order to gain social acceptance (Kandemir and Ozbay, 2009).

However, empathy is not only an individual characteristic but also a construct closely related to self-efficacy beliefs in the context of inclusive education. Empathy enables teachers to understand students’ emotions, systemic barriers, and living conditions (Akgul and Akman, 2025; Aldrup et al., 2022). Teachers with higher levels of SE develop greater awareness of cultural diversity and can reduce anxiety related to intercultural communication in the classroom (Akgul and Akman, 2025). This may be linked to more adaptable and flexible instructional orientations (Yazici et al., 2025). Empathy also enhances positive attitudes toward inclusive education and strengthens teachers’ perceived competence in inclusive classrooms (Jiang et al., 2025). Highly empathic teachers are more likely to support the inclusion of students with diverse learning needs in general education settings (Kudrnáč et al., 2024).

Empathy is also closely related to the quality of teacher–student interaction. Empathic teachers are able to create safe classroom environments in which students feel trust, experience increased motivation, and have their self-confidence supported (De Klerk and De Klerk, 2018). However, the effectiveness of empathy is closely related to teachers’ emotional self-efficacy and their beliefs regarding social dominance (Graziano et al., 2024; Navarro-Mateu et al., 2019). High levels of empathy combined with low emotional regulation skills may increase the risk of burnout and “compassion fatigue.” Therefore, emotional self-efficacy serves as an important balancing factor that transforms empathy into a sustainable tool for inclusive teaching (Graziano et al., 2024).

The relationship between ETI and TEIP is also explained through self-efficacy beliefs. Self-efficacy is defined as an individual’s belief in their own teaching capacity and plays a fundamental role in the development of positive attitudes toward the profession (Eroglu and Unlu, 2015; Kaleli, 2020). It has been found that pre-service teachers with high self-efficacy perceptions demonstrate greater professional commitment and develop more positive attitudes toward teaching (Uyanik, 2016; Incik and Kilic, 2014). In this context, self-efficacy is considered an inseparable component of teacher identity (Nikoçeviq-Kurti, 2021).

The interaction between self-efficacy and professional identity becomes particularly visible in the context of inclusive education. It has been shown that factors such as teaching experience, knowledge of inclusive education policies, pre-service training, and direct contact with individuals with disabilities influence self-efficacy (Wray et al., 2022). In addition, teachers who believe that inclusive education is effective have been found to report higher levels of self-efficacy (Woodcock et al., 2023). These factors are largely intertwined with the development of teacher professional identity.

However, it is also emphasized that the relationship between self-efficacy and attitudes may vary depending on context. Studies conducted with pre-service teachers in pedagogical formation programs have found that this relationship is positive but relatively weak (Ozdemir and Demircioglu, 2016). This finding is explained by variables such as lack of experience and differences across training programs (İpek and Camadan, 2012; Yelpaze and Yakar, 2020). Longitudinal studies also indicate that while self-efficacy increases over time, attitudes do not always change at the same rate (Bumen and Ercan Ozaydin, 2013).

In conclusion, the relationship among ETI, SE, and TEIP demonstrates a multidimensional, dynamic, and experience-based structure. This structure is shaped not only by individual characteristics but also by educational experiences, contextual factors, and professional development processes. Therefore, a high-quality teacher education process should not be limited to knowledge transmission; rather, it should be based on a holistic framework that supports the development of high empathy, strong self-efficacy, and a robust professional identity (Agcam and Babanoglu, 2016; Elaldi and Yerliyurt, 2016).

## 2 Method

### 2.1 Research design

This study employed a quantitative, cross-sectional survey design to examine the relationships among ETI, SE, and TEIP among pre-service teachers. A structural equation modeling (SEM) approach using partial least squares (PLS-SEM) was adopted for data analysis, implemented through SmartPLS 4.0 (Ringle et al., 2024). PLS-SEM was selected over covariance-based SEM (CB-SEM) given the composite-based nature of the constructs, the exploratory-predictive orientation of the study, and the non-normal distribution characteristics observed in the data (Hair et al., 2019). The study follows a two-stage analytical procedure: first evaluating the measurement model for reliability and validity, followed by testing the structural model and hypothesized relationships. Given the cross-sectional and self-report design of the data, the hypothesized pathways in the model were interpreted as statistical associations rather than as evidence of causal or temporal relationships.

### 2.2 Participants and sampling

The study sample consisted of 510 pre-service teachers enrolled in undergraduate teacher education programs at various universities across Turkey. Participants were recruited through a convenience sampling approach during the 2025–2026 academic year. The sample comprised two groups: pre-service early childhood education teachers (*n* = 267, 52.4%) and pre-service primary education teachers (*n* = 243, 47.6%). In total, participants were enrolled across seven universities, reflecting a diverse institutional representation.

The distribution of participants across universities indicated that the majority of the pre-service teachers were enrolled at Hasan Kalyoncu University (*n* = 233, 45.7%). This was followed by Hacettepe University (*n* = 93, 18.2%), Harran University (*n* = 64, 12.5%), Karamanoğlu Mehmetbey University (*n* = 53, 10.4%), Adıyaman University (*n* = 28, 5.5%), Dicle University (*n* = 12, 2.4%), and Gaziantep University (*n* = 1, 0.2%). In addition, 26 responses included incomplete, inaccurate, or unclassifiable university information and were therefore reported as “unclassified/university not reported” (*n* = 26, 5.1%). These cases were retained in the analytical sample because the remaining data provided by these participants were valid and usable for the purposes of the study. Overall, data from 510 pre-service teachers were included in the analysis.

As presented in Table 1, the sample was predominantly female (*n* = 436, 85.5%), consistent with the gender composition typically observed in early childhood and primary education programs in Turkey. In terms of academic year, the sample was distributed across all four undergraduate years, with the largest proportions in the third (28.0%) and fourth (28.2%) years of study.

**Table 1:** Demographic characteristics of participants.

Variable	Early childhood education (*n* = 267)	Primary education (*n* = 243)	Total (*n* = 510)
Gender			
Female	240 (89.9%)	196 (80.7%)	436 (85.5%)
Male	27 (10.1%)	47 (19.3%)	74 (14.5%)
Year of study			
1st year	61 (22.8%)	28 (11.5%)	89 (17.5%)
2nd year	27 (10.1%)	107 (44.0%)	134 (26.3%)
3rd year	75 (28.1%)	68 (28.0%)	143 (28.0%)
4th year	104 (38.9%)	40 (16.5%)	144 (28.2%)

### 2.3 Data collection instruments

Data were collected using three validated self-report scales, all of which had been previously adapted into Turkish. An overview of the instruments is provided in Table 2. All scales used a Likert-type response format, and participants completed the questionnaire battery in a single administration session. The total scale contained 50 items (17 + 15 + 18).

**Table 2:** Overview of data collection instruments.

Scale	Construct	Items	Sub-dimensions	References
Attitude toward teaching profession scale	ETI	17	3	Friesen and Besley (2013); adapted by Arpaci and Bardakci (2015)
Social empathy scale	SE	15	4	Segal et al. (2013); adapted by Bekil (2019)
Teacher efficacy for inclusive practices scale	TEIP	18	3	Sharma et al. (2012); adapted by Tanriverdi and Ozokcu (2018)

#### 2.3.1 Attitude toward teaching profession scale (ATPS)

Pre-service teachers’ ETI was assessed using the ATPS, originally developed by Friesen and Besley (2013) and adapted into Turkish by Arpaci and Bardakci (2015). The scale consists of 17 items organized across three sub-dimensions: Self-Categorization (SC), which captures the extent to which individuals identify themselves as teachers; Confidence in Becoming a Teacher (CBT), which reflects self-assurance regarding one’s readiness for the profession; and Participation as a Teacher (PT), which assesses behavioral involvement in teacher-related activities and roles. Items are rated on a five-point Likert scale ranging from 1 (Strongly Disagree) to 5 (Strongly Agree). Higher scores indicate a stronger sense of early professional identity as a teacher. The Early Teacher Identity measure included items such as “I often doubt if I am the right person to become a teacher.” “I have confidence in my ability to one day be a good teacher”.

#### 2.3.2 Social empathy scale (SES)

SE was measured using the SES, originally developed by Segal et al. (2013) and adapted into Turkish by Bekil (2019). The scale comprises 15 items and encompasses four sub-dimensions: Macro Perspective-Taking (MPT), which reflects the ability to understand systemic and structural factors affecting others’ experiences; Cognitive Empathy (COG), which captures the capacity to understand others’ thoughts and perspectives; Self–Other Awareness (SOA), which measures awareness of the distinction between one’s own and others’ emotional states; and Affective Response (AR), which reflects the emotional reactions elicited by others’ experiences. Responses are provided on a five-point Likert scale (1 = Never, 5 = Always). The Social Empathy measure included items such as “I am aware of other people’s emotions.” “I can explain to others how I am feeling”.

#### 2.3.3 Teacher efficacy for inclusive practices scale (TEIPS)

TEIP was assessed using the TEIPS, developed by Sharma et al. (2012) and adapted into Turkish by Tanriverdi and Ozokcu (2018). The scale consists of 18 items organized into three sub-dimensions: Efficacy in using Inclusive Instruction (EII), which pertains to confidence in employing differentiated and inclusive teaching strategies; Efficacy in Collaboration (EC), which reflects confidence in working collaboratively with families, specialists, and colleagues to support diverse learners; and Efficacy in Managing Behavior (EMB), which captures perceived competence in managing the behavior of students with varying needs in inclusive settings. Items are rated on a six-point Likert scale ranging from 1 (Strongly Disagree) to 6 (Strongly Agree). The Teacher Efficacy for Inclusive Practices Scale included items such as “I can control disruptive behavior in the classroom” “I can accurately gauge student comprehension of what I have taught”.

### 2.4 Data collection procedure

Data were collected between March 16, 2026 and April 2, 2026 using an online survey administered via Google Forms. The questionnaire link was distributed to participants in person during scheduled class sessions at seven universities across Turkey. The researchers shared the survey link directly with students while present in the classroom, ensuring a structured and supervised data collection environment. Participation was voluntary, and all respondents provided informed consent prior to completing the survey.

A total of 1,653 pre-service teachers were invited to participate in the study, and 573 responses were initially received. After excluding 63 incomplete or invalid questionnaires, 510 valid responses were retained for the final analysis, corresponding to a valid response rate of 30.9%. Participants were required to (a) be enrolled in either an early childhood teacher education program or a primary school teacher education program, (b) be actively attending undergraduate courses during the data collection period, and (c) voluntarily agree to participate in the study. To minimize potential coercion associated with classroom-based recruitment, participants were explicitly informed that participation was entirely voluntary and that declining participation would have no academic consequences. Responses were collected anonymously, and no identifying information was requested. Students were also informed that they could discontinue participation at any stage without penalty. Several procedural remedies were implemented to reduce common method bias. Participants were assured of anonymity and confidentiality, informed that there were no right or wrong answers, and encouraged to respond honestly. In addition, because the study relied exclusively on self-report measures, the findings may have been influenced by social desirability bias, particularly for constructs related to empathy and inclusive efficacy.

### 2.5 Data analysis

Data analysis was conducted using SmartPLS 4.0 (Ringle et al., 2024) following the two-stage PLS-SEM procedure recommended by Hair et al. (2019). In the first stage, the measurement model was assessed for indicator reliability (outer loadings ≥ 0.70), internal consistency (Cronbach’s alpha and composite reliability ≥ 0.70), convergent validity (average variance extracted, AVE ≥ 0.50), and discriminant validity using the Fornell–Larcker criterion and the Heterotrait–Monotrait ratio (HTMT < 0.85). In the second stage, the structural model was evaluated by examining the coefficient of determination (*R*^2^), effect sizes (*f*^2^), predictive relevance (*Q*^2^ predict), and model fit (SRMR < 0.08). In addition to statistical significance, the magnitude of the standardized path coefficients (*β*) was interpreted using widely applied SEM benchmarks: *β* < 0.10 = negligible, 0.10–0.29 = small, 0.30–0.49 = moderate, 0.50–0.69 = strong, and ≥ 0.70 = very strong. Hypothesis testing was performed using a bootstrapping procedure with 5,000 subsamples to obtain bias-corrected confidence intervals and *t*-statistics for path coefficients. The mediation hypothesis (H4: ETI → SE → TEIP) was tested by examining the significance of the indirect effect via bootstrapping, with partial mediation inferred when both the direct and indirect effects were statistically significant (*p* < 0.05). Although the terminology of direct, indirect, and mediated paths is used in accordance with SEM conventions, these paths should be interpreted as statistical associations that are consistent with the proposed theoretical model, rather than as causal mechanisms.

## 3 Results

This section presents the findings of the SEM analysis conducted using SmartPLS 4.0 to examine the relationships among ETI, SE, and TEIP among pre-service primary and early childhood education teachers. The analysis followed a two-stage approach: first, the measurement model was evaluated for reliability and validity; second, the structural model was tested to examine the proposed hypotheses.

### 3.1 Measurement model assessment

Prior to testing the structural model, the psychometric properties of the measurement model were evaluated to confirm that all constructs demonstrated adequate reliability and validity. This evaluation included assessments of indicator loadings, convergent validity, and discriminant validity.

#### 3.1.1 Indicator loadings and descriptive statistics

Table 3 presents the outer loadings and descriptive statistics (mean, excess kurtosis, and skewness) for all items included in the three constructs. All item loadings met or exceeded the recommended threshold of 0.70 (Hair et al., 2019). The highest loadings were observed in the TEIP construct, particularly within the EC (EC4 = 0.908) and EMB (EMB4 = 0.933) sub-dimensions, suggesting strong item-construct relationships. Regarding distributional properties, most items exhibited negative skewness, indicating a tendency toward higher ratings across all constructs. Given that PLS-SEM does not assume multivariate normality, skewness and kurtosis statistics were reported for descriptive purposes only.

**Table 3:** Indicator loadings and descriptive statistics.

Construct	Sub-dimension	Item	Loading	Mean	Excess kurtosis	Skewness
ETI	SC	SC1	0.841	3.965	0.880	−1.031
		SC2	0.843	4.049	1.224	−1.146
		SC3	0.862	3.853	0.092	−0.681
		SC4	0.715	3.837	−0.126	−0.851
		SC5	0.833	3.925	0.232	−0.843
	CBT	CBT1	0.736	3.910	−0.174	−0.787
		CBT2	0.783	4.241	1.903	−1.394
		CBT3	0.785	3.696	−0.565	−0.514
		CBT4	0.835	4.102	1.682	−1.263
		CBT5	0.835	4.276	2.834	−1.614
		CBT6	0.822	4.078	1.172	−1.121
	PT	PT1	0.784	3.886	0.657	−1.014
		PT2	0.706	3.486	−0.402	−0.449
		PT3	0.831	4.239	2.527	−1.539
		PT4	0.753	3.822	0.233	−0.789
		PT5	0.892	4.339	3.715	−1.791
		PT6	0.897	4.335	2.977	−1.619
SE	MPT	MPT1	0.756	3.890	0.960	−0.738
		MPT2	0.793	4.096	0.765	−0.942
		MPT3	0.785	3.655	−0.010	−0.694
		MPT4	0.806	4.161	1.067	−0.924
		MPT5	0.766	3.914	0.499	−0.882
	COG	COG1	0.775	3.827	0.561	−0.644
		COG2	0.854	4.149	1.560	−1.033
		COG3	0.831	3.914	0.294	−0.638
		COG4	0.846	3.992	1.477	−1.035
	SOA	SOA1	0.799	3.886	0.766	−0.679
		SOA2	0.881	4.118	2.223	−1.220
		SOA3	0.794	3.653	−0.194	−0.553
	AR	AR1	0.875	4.355	2.171	−1.372
		AR2	0.888	4.239	1.353	−1.065
		AR3	0.814	4.139	0.804	−0.965
TEIP	EII	EII1	0.908	4.771	2.444	−1.383
		EII2	0.761	4.465	1.041	−1.034
		EII3	0.855	4.578	1.004	−0.996
		EII4	0.935	4.929	2.588	−1.473
		EII5	0.913	4.902	2.440	−1.455
		EII6	0.882	4.827	1.845	−1.360
	EC	EC1	0.893	4.775	2.346	−1.398
		EC2	0.897	4.816	2.578	−1.450
		EC3	0.897	4.676	1.534	−1.207
		EC4	0.908	4.833	1.787	−1.306
		EC5	0.873	4.741	1.384	−1.207
		EC6	0.840	4.565	0.847	−1.030
	EMB	EMB1	0.849	4.567	0.792	−0.978
		EMB2	0.876	4.482	1.060	−0.917
		EMB3	0.923	4.616	1.602	−1.152
		EMB4	0.933	4.624	1.558	−1.173
		EMB5	0.906	4.888	2.573	−1.450
		EMB6	0.837	4.424	0.851	−0.982

ETI, Early Teacher Identity; SC, Self-Categorization; CBT, Confidence in Becoming a Teacher; PT, Participation as a Teacher; SE, Social Empathy; MPT, Macro Perspective-Taking; COG, Cognitive Empathy; SOA, Self–Other Awareness; AR, Affective Response; TEIP, Teacher Efficacy for Inclusive Practices; EII, Efficacy in using Inclusive Instruction; EC, Efficacy in Collaboration; EMB, Efficacy in Managing Behavior. CA, Cronbach’s Alpha.

#### 3.1.2 Reliability and convergent validity

Table 4 presents the reliability and convergent validity indicators for all constructs and their respective sub-dimensions. Internal consistency reliability was assessed using Cronbach’s alpha (CA), Dijkstra-Henseler’s rho_A, and composite reliability (CR). All constructs demonstrated excellent reliability, with CA values ranging from 0.706 (SOA) to 0.980 (TEIP), rho_A values consistently above 0.70, and CR values exceeding the recommended threshold of 0.70 for all constructs (Hair et al., 2019).

**Table 4:** Reliability and convergent validity indicators.

Construct/sub-dimension	CA	rho_A	CR	AVE
ETI	0.929	0.946	0.939	0.592
SC	0.841	0.872	0.889	0.623
CBT	0.791	0.847	0.846	0.590
PT	0.870	0.890	0.904	0.616
SE	0.934	0.938	0.942	0.524
MPT	0.820	0.827	0.874	0.581
COG	0.846	0.848	0.896	0.684
SOA	0.706	0.733	0.837	0.633
AR	0.823	0.826	0.895	0.739
TEIP	0.980	0.981	0.981	0.745
EII	0.939	0.944	0.952	0.770
EC	0.944	0.946	0.956	0.783
EMB	0.946	0.948	0.957	0.788

Convergent validity was assessed through the average variance extracted (AVE). The AVE values for all constructs met or exceeded the recommended criterion of 0.50 (Fornell and Larcker, 1981), with values ranging from 0.590 (CBT) to 0.788 (EMB). All AVE values met or exceeded the recommended criterion of 0.50, confirming satisfactory convergent validity across all sub-dimensions.

#### 3.1.3 Discriminant validity

Discriminant validity was examined using two criteria: the Fornell-Larcker criterion and the Heterotrait-Monotrait ratio (HTMT). As shown in Table 5, the Fornell-Larcker criterion was satisfied for all constructs; the square root of each construct’s AVE (displayed on the diagonal) exceeded its correlations with all other constructs. Specifically, the square root of the AVE for ETI (0.701), SE (0.724), and TEIP (0.863) were all greater than the inter-construct correlations, indicating that each construct shares more variance with its own indicators than with those of other constructs (Fornell and Larcker, 1981).

**Table 5:** Fornell–Larcker criterion and HTMT ratios for discriminant validity.

Variable	Fornell–Larcker criterion			HTMT		
	ETI	SE	TEIP	ETI	SE	TEIP
ETI	**0.701**			–		
SE	0.559	**0.724**		0.591	–	
TEIP	0.594	0.567	**0.863**	0.611	0.591	–

Bold diagonal values are √AVE.

The HTMT ratios further confirmed discriminant validity. All HTMT values were well below the conservative threshold of 0.85 (Henseler et al., 2015), with the highest value observed between ETI and TEIP (HTMT = 0.611). These results collectively provide strong evidence for the discriminant validity of the measurement model.

### 3.2 Model fit

The overall model fit was evaluated using the Standardized Root Mean Square Residual (SRMR) and the unweighted least squares discrepancy (d_ULS), as recommended for PLS-SEM (Henseler et al., 2016). As presented in Table 6, the SRMR value for the saturated model was 0.074 and for the estimated model was 0.076, both below the recommended threshold of 0.08 (Hu and Bentler, 1999), indicating an acceptable model fit. The d_ULS values were 27.711 and 28.864 for the saturated and estimated models, respectively. Chi-square and NFI indices were not available due to the nature of PLS-SEM estimation. Overall, these fit statistics suggest that the proposed model adequately represents the data.

**Table 6:** Model fit summary.

Index	Saturated model	Estimated model
SRMR	0.074	0.076
d_ULS	27.711	28.864
d_G	n/a	n/a
Chi-square	n/a	n/a
NFI	n/a	n/a

### 3.3 Structural model results

Following the confirmation of measurement model adequacy, the structural model was evaluated to examine the explanatory power, predictive relevance, and effect sizes of the proposed relationships. Table 7 summarizes the key structural model indicators.

**Table 7:** Structural model indicators.

Variable/relationship	*R* ^2^	*Q*^2^ predict	RMSE	MAE	*f* ^2^	VIF
TEIP	0.433	0.348	0.814	0.570		
SE	0.312	0.308	0.838	0.626		
SE → TEIP					0.142	1.454
ETI → TEIP					0.196	1.454
ETI → SE					0.454	1.000

The model explained 43.3% of the variance in TEIP (*R*^2^ = 0.433) and 31.2% of the variance in SE (*R*^2^ = 0.312), both of which represent moderate to substantial explanatory power according to Cohen’s, (1988) benchmarks. Predictive relevance was assessed using the *Q*^2^ predict statistic. Values of *Q*^2^ predict > 0 confirm that the model has predictive relevance for both TEIP (*Q*^2^ = 0.348) and SE (*Q*^2^ = 0.308) (Shmueli et al., 2019). Root Mean Square Error (RMSE) and Mean Absolute Error (MAE) values were within acceptable ranges, further supporting the model’s predictive accuracy.

Effect sizes (*f*^2^) were calculated for each path in the structural model. ETI showed the largest effect size on SE (*f*^2^ = 0.454), which is classified as a large effect according to Cohen’s, (1988) guidelines (*f*^2^ ≥ 0.35). The direct effect of ETI on TEIP (*f*^2^ = 0.196) and the indirect effect mediated by SE (*f*^2^ = 0.142) were both classified as small-to-medium effects. Variance Inflation Factor (VIF) values for all paths were below the threshold of 3.3 (Kock, 2015), confirming the absence of multicollinearity in the structural model.

### 3.4 Hypothesis testing

**Figure 2:**
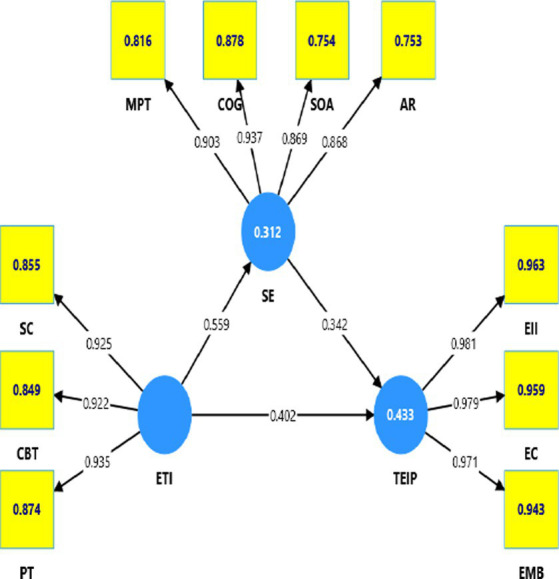
Path analysis of the measurement model.

**Figure 3:**
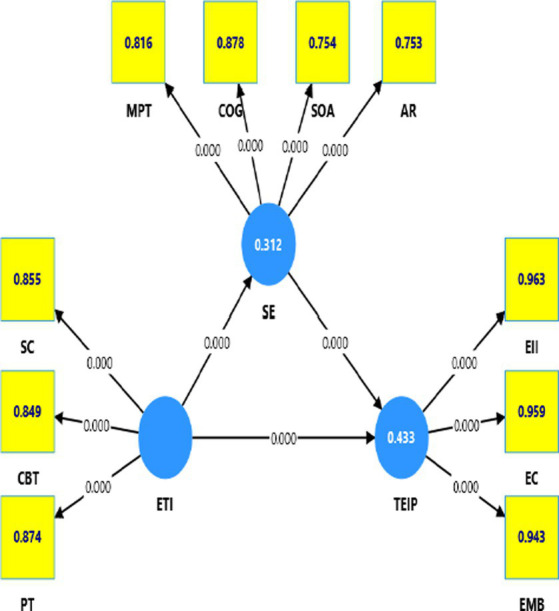
Bootstrapping procedure for the measurement model.

The hypothesized structural paths were tested using a bootstrapping procedure with 5,000 resamples to obtain stable standard errors and confidence intervals (Hair et al., 2019). Table 8 presents the results of the hypothesis tests. Figures 2, 3 display the path diagram of the measurement model and the bootstrapping output, respectively.

**Table 8:** Structural equation modeling results: hypothesis testing.

Hypothesis	Relationship	Original sample	Sample mean	*t-*statistic	*p*	Decision
H1	ETI → SE	0.559	0.559	13.971	0.000	Supported
H2	SE → TEIP	0.342	0.343	6.049	0.000	Supported
H3	ETI → TEIP	0.402	0.401	6.995	0.000	Supported
H4 (indirect)	ETI → SE → TEIP	0.191	0.192	5.062	0.000	Supported

The hypothesized relationships were tested using the bootstrapping procedure with 5,000 subsamples, as recommended for PLS-SEM (Hair et al., 2019). Table 8 presents the path coefficients, sample means, *t*-statistics, and *p*-values for each hypothesis.

H1 proposed a positive association between ETI and SE among pre-service teachers. The results revealed a significant and positive path coefficient (*β* = 0.559, *t* = 13.971, *p* < 0.001), providing strong support for H1. In terms of effect size, this coefficient reflects a strong association according to widely applied SEM benchmarks (0.50–0.69 = strong). This finding indicates that higher levels of ETI are associated with greater SE among primary and early childhood education teacher candidates.

H2 proposed a positive association between SE and TEIP. This relationship was also statistically significant (*β* = 0.342, *t* = 6.049, *p* < 0.001), supporting H2. In terms of effect size, this path coefficient reflects a moderate association (0.30–0.49 = moderate). Pre-service teachers who report higher SE demonstrate greater efficacy for inclusive practices in their teaching beliefs and intentions.

H3 examined the direct effect of ETI on TEIP. The results confirmed a significant positive relationship (*β* = 0.402, *t* = 6.995, *p* < 0.001), providing support for H3. In terms of effect size, this coefficient reflects a moderate association (0.30–0.49 = moderate). This suggests that ETI has a significant direct statistical association with TEIP, even when SE is included in the model.

H4 tested the indirect (mediating) effect of SE in the relationship between ETI and TEIP. The bootstrapped indirect effect was significant (*β* = 0.191, *t* = 5.062, *p* < 0.001), confirming that SE partially mediates the ETI–TEIP relationship. In terms of effect size, the magnitude of this indirect effect is small (0.10–0.29 = small). The simultaneous significance of both direct (H3) and indirect effects (H4) indicates partial mediation, suggesting that SE represents a statistically significant but not exclusive indirect pathway linking ETI and TEIP in the proposed model.

Taken together, all four hypotheses were supported at *p* < 0.001. Taken together, the findings indicate that ETI is strongly and positively associated with SE and moderately and positively associated with TEIP, and that SE accounts for a small but statistically significant partial indirect association between ETI and TEIP among pre-service teachers.

## 4 Discussion and conclusion

This study investigated the relationships among ETI, SE, and TEIP among pre-service teachers. The findings provide empirical support for a theoretically grounded relational model in which ETI is directly associated with TEIP and indirectly associated with TEIP through SE. Given the cross-sectional and self-report design, these findings should be interpreted as associations that are consistent with the proposed model rather than as evidence of causal or developmental processes.

The first key finding indicates that ETI is significantly associated with SE (H1). This result suggests that identity-related orientations in teacher education appear to be closely linked with socio-emotional dispositions such as SE. In this sense, pre-service teachers who report stronger identification with the teaching role also tend to report higher levels of capacity to understand others’ emotions, perspectives, and social conditions. This finding is consistent with previous research emphasizing that teacher identity is not limited to professional role perception but is deeply embedded in interpersonal and emotional dispositions (Ince and Coskuner, 2025). Similarly, Chen et al. (2025) and Sun et al. (2025) highlight that teacher identity and empathy appear to be closely related within professional development contexts, indicating a bidirectional rather than unidirectional relationship. Given the relational nature of teacher identity, the significant association between ETI and SE can be understood as more than just a cognitive self-perception of being a future teacher (Akkerman and Meijer, 2011; Beauchamp and Thomas, 2009; Beijaard et al., 2004). Teacher identity is constructed through interactions, institutional expectations, professional values, and reflective meaning-making processes (Beauchamp and Thomas, 2009; Kelchtermans, 2009; Trent, 2010; Yuan and Lee, 2015). From this perspective, pre-service teachers with a stronger sense of early teacher identity may be more likely to view themselves as responsible, responsive, and relational professionals (Beauchamp and Thomas, 2009; Villegas and Lucas, 2002). This aligns with identity-based approaches, which argue that becoming a teacher involves not only acquiring pedagogical knowledge but also developing sensitivity to learners’ diverse experiences, needs, and social conditions (Gay, 2002; Mills and Ballantyne, 2010; Villegas and Lucas, 2002). In this study, the emphasis on early teacher identity is especially significant, as pre-service teachers are still negotiating their professional self-understanding. During this formative stage, identity-related beliefs are likely to be particularly sensitive to influences such as coursework, field experiences, exposure to diversity, and models of inclusive teaching.

The second finding shows that SE is positively associated with TEIP (H2). This result is consistent with the growing body of literature emphasizing empathy as a fundamental socio-emotional competence in inclusive education. Empathic teachers are more capable of recognizing diverse learning needs, understanding structural barriers, and designing adaptive instructional strategies (Akgul and Akman, 2025; Aldrup et al., 2022). Moreover, empirical evidence indicates that empathy is associated with teachers’ ability to create supportive, differentiated, and motivating learning environments, and may be linked to higher levels of student engagement and participation (De Klerk and De Klerk, 2018; Gómez-Arizaga et al., 2016). From this perspective, SE may function as a cognitive-affective link between understanding diversity and perceived readiness for inclusive instructional practices.

The positive association between SE and TEIP indicates that pre-service teachers’ perceived efficacy for inclusive practices may be related not only to pedagogical knowledge but also to their ability to understand learners within broader social and structural contexts (Jiang et al., 2025; Sharma et al., 2012; Woodcock et al., 2022). Unlike interpersonal empathy, social empathy entails an awareness of systemic barriers, social inequalities, and contextual factors that influence students’ educational experiences (Segal, 2011; Segal et al., 2012; Wagaman, 2011). Consequently, pre-service teachers with higher SE may be more likely to view inclusive education as an issue of participation, equity, and responsiveness, rather than simply as a technical process of adapting instruction to address individual deficits (Ainscow, 2020; Florian, 2014; Florian and Black-Hawkins, 2011; Sharma et al., 2021).

The third finding reveals that ETI was directly associated with TEIP, independent of SE (H3). This suggests that identity-related beliefs may be closely connected to professional confidence in inclusive settings. Accordingly, teacher identity can be conceptualized as a core psychological structure related to competence, responsibility, and professional agency. Consistent with this interpretation, previous studies have shown that self-efficacy is not only related to teaching attitudes but also an integral component of professional identity formation (Nikoçeviq-Kurti, 2021). This indicates that early identity-related orientations may be linked to a stronger sense of professional agency and perceived capability for inclusive teaching.

The direct association between ETI and TEIP supports research indicating that teacher identity and self-efficacy are closely linked in the process of teacher development (Lamote and Engels, 2010; Nikoçeviq-Kurti, 2021; Rodrigues and Mogarro, 2019). In the context of inclusive education, efficacy beliefs extend beyond general teaching confidence to include perceived abilities to adapt instruction, collaborate with families and professionals, and manage diverse classroom needs (Sharma et al., 2012). As such, pre-service teachers with a stronger early identification with the teaching profession may also report greater confidence in fulfilling the complex responsibilities of inclusive classrooms (Loreman et al., 2013; Sharma et al., 2021). This is consistent with studies showing that inclusive teaching efficacy is associated with teacher education experiences, knowledge of inclusion policies, direct contact with individuals with disabilities, and practicum opportunities (Loreman et al., 2013; Peebles and Mendaglio, 2014; Sokal et al., 2013; Song et al., 2019).

The fourth finding indicates a significant indirect association between ETI and TEIP through SE (H4). The indirect association suggests that the link between ETI and TEIP may be partly accounted for by SE. In other words, pre-service teachers who report stronger ETI also tend to report higher SE, which is in turn associated with stronger perceived efficacy for inclusive practices. However, this pattern should not be interpreted as evidence that ETI causes SE or that SE causes TEIP. This mediating structure is consistent with recent theoretical and empirical studies emphasizing multi-path and sequential mediation models in teacher development (Jiang et al., 2025). Empathy has been shown to mediate various educational outcomes, including inclusive attitudes, multicultural competence, and beliefs in social justice (Hut et al., 2025; Sookyoung, 2017). The present findings extend this literature by suggesting that ETI may be considered an earlier-positioned construct within the proposed relational model. This finding adds to the literature by suggesting that the relationship between identity-related orientations and inclusive efficacy may be partially socio-emotional in nature (Beauchamp and Thomas, 2009; Izadinia, 2013; Jiang et al., 2025). While previous studies have typically examined teacher identity, empathy, and inclusive efficacy as separate constructs, the present study integrates these variables within a single model (Rodrigues and Mogarro, 2019; Segal, 2011; Sharma et al., 2012). The results suggest that social empathy may serve as a key link connecting pre-service teachers’ professional self-understanding with their perceived readiness for inclusive practice (Akkerman and Meijer, 2011; Jiang et al., 2025; Segal et al., 2012; Sharma et al., 2012).

Importantly, the literature suggests that these relationships are not static but context-dependent and influenced by career stage and experiential learning. For instance, studies indicate that teacher self-efficacy may decline during the transition from pre-service education to early career teaching (Dignath et al., 2022; Mintz et al., 2020). This highlights the vulnerability of efficacy beliefs at the point of professional entry and underscores the importance of sustainable identity and empathy development. Similarly, practicum experiences and direct interaction with diverse learners have been identified as critical factors shaping both empathy and efficacy in inclusive education contexts (Coates et al., 2020; Specht and Metsala, 2018). These findings support the interpretation that experiential learning may be associated with variations in the identity–empathy–efficacy pathway.

The findings of this study are also consistent with broader evidence in teacher education research, which has consistently reported positive relationships among professional attitudes, self-efficacy, and teaching commitment (Ataş Akdemir, 2018; Emre and Unsal, 2017; İspir and Yildiz, 2023). Similar to the present model, self-efficacy has been identified as a partial mediator between professional attitudes and teaching commitment (İspir and Yildiz, 2023). This cyclical relationship suggests that changes in one domain may be related to changes in the others, suggesting a dynamic relational pattern in teachers’ professional growth.

At the international level, large-scale evidence is consistent with the findings of this study. A comprehensive synthesis of 25 studies involving more than 47,000 teachers across over 40 countries indicates a strong positive relationship between socio-emotional constructs and TEIP (Dignath et al., 2022). Moreover, Graziano et al. (2024) found that empathy significantly associated with inclusive teaching self-efficacy, particularly when emotional regulation capacities are high. These findings highlight that empathy is not merely an emotional disposition but also a functional competence that may be relevant to perceived professional effectiveness in inclusive classrooms.

Beyond its instructional implications, SE also carries broader societal significance. It is associated with social responsibility, awareness of social justice, and prosocial behavior (Segal et al., 2012; Silke et al., 2024). In educational settings, teachers play a crucial role in fostering these outcomes by creating inclusive, emotionally responsive, and socially supportive classroom environments. Such environments reduce aggression and bullying while enhancing peer relationships and socio-emotional development (Silke et al., 2024). Therefore, SE should be conceptualized not only as a pedagogical skill but also as a civic and ethical competence essential for democratic societies.

In summary, the findings suggest that ETI, SE, and TEIP are interrelated constructs within the proposed theoretical model. Rather than indicating a causal developmental sequence, the results show a pattern of associations in which SE statistically accounts for part of the relationship between ETI and TEIP. This relational pattern highlights the value of considering identity-related, socio-emotional, and efficacy-related dimensions together in teacher education research on inclusive education. This multi-layered structure underscores the importance of viewing teacher development as a holistic process in which identity-related, socio-emotional, and professional competence dimensions are considered together within the context of inclusive education. This study extends previous research in several important respects. First, it brings early teacher identity, social empathy, and inclusive teaching efficacy together within a single theoretically grounded model, thereby addressing an important gap in the literature where these constructs have often been examined separately. Second, the study highlights the specific relevance of social empathy, rather than empathy understood only as an interpersonal disposition, for explaining pre-service teachers’ perceived efficacy for inclusive practices. This distinction is particularly important because social empathy includes awareness of systemic barriers, social inequalities, and contextual factors that are related to learners’ educational experiences, all of which are central to the philosophy and practice of inclusive education. Third, by focusing on pre-service early childhood and primary education teachers, the study contributes to a deeper understanding of how identity-related and socio-emotional orientations are associated with inclusive efficacy before teachers enter the profession.

## 5 Limitations

First, the cross-sectional nature of the study limits the ability to draw causal inferences among the variables. Although the proposed relationships were theoretically grounded and statistically supported, the findings should be interpreted as associative rather than causal.

Second, this study employed self-report instruments to collect data from pre-service teachers. In this regard, the possibility of social desirability bias in participants’ responses constitutes a potential limitation of the study. Self-report measures, by nature, may introduce common method bias and may not fully capture actual behaviors or competencies in inclusive teaching contexts.

Although affective empathy is highly relevant in educational settings, particularly for teacher–student relationships and caring pedagogical responses (Meyers et al., 2019), the present study focuses specifically on social empathy because the selected measure captures cognitive-contextual understanding of others’ lived experiences rather than emotional sharing or affective resonance (Segal, 2011; Segal et al., 2013). Therefore, the findings should be interpreted in relation to the social-cognitive and contextual dimensions of empathy rather than affective empathy.

In addition, the study relied exclusively on questionnaire data and did not include observational, behavioral, or performance-based measures. Future studies may benefit from incorporating classroom observations, peer evaluations, or longitudinal behavioral indicators to provide a more comprehensive understanding of the constructs examined.

The use of convenience sampling also limits the representativeness of the sample and may restrict the generalizability of the findings to all pre-service teachers in Turkey.

In addition, the gender distribution of the sample represents another limitation. Approximately 15% of the participants were male, while the majority were female. Considering that previous research suggests that socio-emotional variables may vary according to gender, this imbalance in the sample may have influenced the generalizability of the findings across gender groups.

Therefore, the findings should be interpreted with caution when generalized to different cultural contexts, educational systems, or professional groups.

## 6 Recommendations

*Strengthening early interventions:* The findings of this study indicate that ETI has a strong direct association with SE (H1) and a moderate direct association with TEIP (H3). Based on this, it is recommended that pre-service teacher education programs provide structured opportunities for earlier and more sustained field-based experiences. Such experiences should enable pre-service teachers to interact more frequently with children and to actively engage in identity-building processes throughout their training, rather than limiting practical exposure to the final year.

*Integrating SE into the curriculum:* This study identified SE as a bridging construct between teacher identity development and inclusive teaching efficacy. Therefore, explicit learning outcomes related to the development of SE skills could be systematically integrated into undergraduate teacher education curricula. This integration may support the development of both socio-emotional awareness and inclusive pedagogical competencies.

*Further exploration through qualitative and mixed methods designs:* As this study was designed using a quantitative approach, it captures relational patterns among variables but does not fully explain underlying mechanisms. Future research incorporating qualitative methods such as interviews and classroom observations, or employing mixed-method designs, may provide deeper insights into the theoretical processes linking ETI, SE, and TEIP.

*Diversification of study groups across teaching fields:* This study was conducted with pre-service preschool and primary school teachers. Future research may include participants from different teaching fields such as special education, language education, or subject-specific disciplines. This would allow for more nuanced, field-based comparisons and more differentiated interpretations of the relationships among variables.

*Longitudinal research designs:* This study reflects a cross-sectional snapshot of pre-service teachers at a single point in time. Future studies should examine the development of ETI, SE, and TEIP longitudinally, from the beginning of undergraduate education through the transition into the teaching profession. Such designs would enable a more comprehensive understanding of how these constructs evolve over time and interact across different career stages.

## Data Availability

The raw data supporting the conclusions of this article will be made available by the authors, without undue reservation.
